# Genomic prediction of switchgrass winter survivorship across diverse lowland populations

**DOI:** 10.1093/g3journal/jkad014

**Published:** 2023-01-17

**Authors:** Neal W Tilhou, Hari P Poudel, John Lovell, Sujan Mamidi, Jeremy Schmutz, Christopher Daum, Matthew Zane, Yuko Yoshinaga, Anna Lipzen, Michael D Casler

**Affiliations:** Department of Agronomy, University of Wisconsin, 1575 Linden Dr, Madison, WI 53706, USA; Lethbridge Research and Development Centre, Agriculture and Agri-Food Canada, Lethbridge, AB, T1J 4B1 Canada; Genome Sequencing Center, HudsonAlpha Institute for Biotechnology, Huntsville, AL 35806, USA; Genome Sequencing Center, HudsonAlpha Institute for Biotechnology, Huntsville, AL 35806, USA; Genome Sequencing Center, HudsonAlpha Institute for Biotechnology, Huntsville, AL 35806, USA; Department of Energy Joint Genome Institute, Lawrence Berkeley National Laboratory, Lawrence, CA 94704, USA; Department of Energy Joint Genome Institute, Lawrence Berkeley National Laboratory, Lawrence, CA 94704, USA; Department of Energy Joint Genome Institute, Lawrence Berkeley National Laboratory, Lawrence, CA 94704, USA; Department of Energy Joint Genome Institute, Lawrence Berkeley National Laboratory, Lawrence, CA 94704, USA; Department of Agronomy, University of Wisconsin, 1575 Linden Dr, Madison, WI 53706, USA

**Keywords:** switchgrass, grass breeding, genomic prediction, winter survival, flowering time, climate change

## Abstract

In the North-Central United States, lowland ecotype switchgrass can increase yield by up to 50% compared with locally adapted but early flowering cultivars. However, lowland ecotypes are not winter tolerant. The mechanism for winter damage is unknown but previously has been associated with late flowering time. This study investigated heading date (measured for two years) and winter survivorship (measured for three years) in a multi-generation population generated from two winter-hardy lowland individuals and diverse southern lowland populations. Sequencing data (311,776 markers) from 1,306 individuals were used to evaluate genome-wide trait prediction through cross-validation and progeny prediction (n = 52). Genetic variance for heading date and winter survivorship was additive with high narrow-sense heritability (0.64 and 0.71, respectively) and reliability (0.68 and 0.76, respectively). The initial negative correlation between winter survivorship and heading date degraded across generations (F_1_  *r* = −0.43, pseudo-F_2_  *r* = −0.28, pseudo-F_2_ progeny *r* = −0.15). Within-family predictive ability was moderately high for heading date and winter survivorship (0.53 and 0.52, respectively). A multi-trait model did not improve predictive ability for either trait. Progeny predictive ability was 0.71 for winter survivorship and 0.53 for heading date. These results suggest that lowland ecotype populations can obtain sufficient survival rates in the northern United States with two or three cycles of effective selection. Despite accurate genomic prediction, naturally occurring winter mortality successfully isolated winter tolerant genotypes and appears to be an efficient method to develop high-yielding, cold-tolerant switchgrass cultivars.

## Introduction

The perennial bunchgrass switchgrass (*Panicum virgatum* L.) is undergoing breeding for improved agronomic performance as a biomass crop (Sanderson *et al.* 2006; Bhandari *et al.* 2015). Commercial adoption of switchgrass is currently limited by insufficient yield performance and lack of robust markets (Dumortier *et al.* 2017; Brandes *et al.* 2018). Breeding efforts in switchgrass have improved yield through multiple routes and within multiple switchgrass ecotypes (Casler and Vogel 2014; Casler *et al.* 2018). One strategy for increasing yield in the north-central United States is through adoption of populations from the southern United States, broadly referred to as lowland ecotypes (Poudel *et al.* 2019a, 2020). Unimproved southern populations are capable of a 50% increase in biomass yield relative to northern-adapted populations when grown in northern regions (Poudel *et al.* 2020). This effect is due to late flowering traits which are common in the southern United States (Lowry *et al.* 2019). Late flowering switchgrass populations have longer periods of vegetative growth which result in greater biomass accumulation (Schwartz *et al.* 2010; Schwartz and Amasino 2013; Casler 2019). Unfortunately, late flowering populations also suffer from high levels of winter mortality (>90%) in northern environments (Poudel *et al.* 2019a). Fortunately, heritable variation in winter hardiness exists in many switchgrass gene pools including southern lowlands (Lovell *et al.* 2021). Breeding progress has been reported for increased winter survivorship in populations through recurrent natural winter mortality, but molecular breeding methods could accelerate selection for winter survival (Poudel *et al.* 2020).

There are multiple potential pathways that could contribute to winter damage and mortality (Sarath *et al.* 2014; Peixoto and Sage 2016; Poudel *et al.* 2019b). For example, perennial plant species require hardening periods during fall to obtain cold hardiness (Sarath *et al.* 2014). A reduced hardening period due to later initiation could result in plant mortality and has been observed in lowland ecotypes (Palmer *et al.* 2014). Alternatively, loss of winter hardness (de-acclimatization) during a short winter or spring warming event could result in damage. Peixoto and Sage (2016) observed differential de-acclimatization in response to simulated spring warming followed by refreezing in *Miscanthus* cultivars. Another possible route is through root mortality, since lowland genotypes produce relatively coarse and long-lived roots compared with northern-adapted ecotypes (Chen *et al.* 2021). Roots produced by these lowland genotypes could be more prone to damage and slow to recover during harsh northern winters. Lastly, mid-winter minimum temperatures or anoxia due to ice cover could also induce mortality. The presence of multiple potential winter stressors both hinders the creation of robust cold tolerance assays and reduces the efficacy of single-year winter selection events.

Likewise, it is likely that there are multiple genetic mechanisms for winter survivorship. The lowland ecotype contains high diversity and multiple differentiated sub-populations (Evans *et al.* 2018). After undergoing multiple cycles of selection for winter survivorship, populations originating from a wide geographic region were all able to obtain greater than 50% winter survivorship (Poudel *et al.* 2020). However, a genetic association study of lowland survivors found few consistent genetic regions under selection across populations (Poudel *et al.* 2021). If multiple mechanisms for winter survival exist in lowland switchgrass, further research may reveal which germplasm sources or loci are the most advantageous to long-term yield gain. For example, many populations collected from the eastern United States were defined as the coastal ecotype and are capable of winter survival in the north-central United States (Poudel *et al.* 2019a; Lovell *et al.* 2021). However, these populations often flower up to a month earlier than the lowland ecotype, a characteristic which limits their use for biomass production (Casler 2019; Poudel *et al.* 2020). Initial observations suggest genetic linkage between flowering time and winter survival which could the limit of yield of northern-adapted lowland switchgrass populations (Schwartz and Amasino 2013; Poudel *et al.* 2020). Genetic correlations between flowering time and winter survival could be due to the natural history of the species (i. e. population structure; Lovell *et al.* 2021) or due to physical linkage of loci influencing each trait within the genome. The latter linkage could be due to physically close loci (linkage disequilibrium), or loci which impact both traits (pleiotropy). Determining if winter survivorship and flowering time are tightly linked can provide valuable information for determining which switchgrass sub-populations are the most promising for future breeding progress.

This study investigated the reliability and genetic determinants of winter survivorship within multiple lowland germplasm sources by constructing a multi-generation pedigree focused on crosses of two individuals with strong winter survival and a diverse group of southern lowland individuals. The dataset was used as training data for genome-wide predictions which were evaluated using cross-validation and through prediction of progeny individuals. Last, individuals grown from bulked progeny seed from this experiment were evaluated for yield and compared to other switchgrass lines from populations under selection.

## Materials and methods

### Germplasm and experimental design

In 2016, initial crosses were carried out by bagging inflorescences of switchgrass ramets in a greenhouse. The majority of crosses occurred between a diverse group of southern genotypes (n = 57) from lowland populations and two lowland genotypes that showed strong winter survivorship as multiple clonal replicates over 6 winters near Arlington, WI. There were also a limited number of crosses between southern genotypes which had not been evaluated for winter survivorship. The winter tolerant genotypes are referred to as Tolerant 1 and Tolerant 2, and they originated from an unknown population originally collected in North Carolina, South Carolina or northern Florida (Timothy DH, personal communication). Collectively, the individuals used for initial crosses will be referred to as Founders. Crosses resulted in 2,058 individuals unevenly distributed across 29 unique F_1_ families. The number of individuals per family was the result of variable seed quantity and viability. During the following year, a set of pseudo-F_2_ families were generated by crossing randomly selected siblings within F_1_ families. This resulted in 1,039 pseudo-F_2_ individuals unevenly distributed among 20 full-sib pseudo-F_2_ families. Some pseudo-F_2_ families were generated from pairs of siblings within an F_1_ family, so only 10 F_1_ families were represented in the pseudo-F_2_ families.

Among controlled greenhouse crosses, the success rate for initial crosses among Founder individuals was 71%, with success defined as resulting in at least one progeny seedling from a parent (mean 36 seedlings per successful cross). Within F_1_ sibling matings, used to generate pseudo-F_2_ families, the success rate was 20%, but with a mean of 74 seedlings generated per successful cross parent.

All Founder individuals and F_1_ parents of pseudo-F_2_ families were maintained in a greenhouse and divided into vegetative replicates by dividing crowns. In July 2018, a completely randomized spaced plant nursery was planted with 195 genotypes. The spaced plants were genotypes maintained in 12-plant rows with 0.7 m between and 0.7 m within rows. Weeds were controlled between individuals genotype crowns using roto-tilling and occasional hand weeding. The nursery contained a minimum of two vegetative replicates per individual. In 2019, vegetative replicates reserved from Founder individuals and F_1_ parents of pseudo-F_2_ families were used to replace individuals that were lost to winterkill in the spaced plant nursery during the first winter.

In addition, an unreplicated, stratified by genotype spaced row nursery (unique genotypes planted with 0.7 m between rows and 0.3 m within rows) was established of the F_1_ families and pseudo-F_2_ families in the spring of 2018. Each row contained 10 unique genotypes. In the summer of 2018, heavy rain and standing water in sections of the nursery and resulted in uneven and poor plant vigor. To account for establishment damage that was unrelated to winter survival, fall vigor ratings were made on a scale of 0 to 5 during September in 2018 and 2019. Fall vigor was then used as a covariate for the subsequent spring vigor scores. A fall vigor score of 5 indicated a healthy switchgrass plant and zero indicated a deceased plant.

Winter survivorship scores and heading date was measured for each individual in both nurseries during 2019, 2020, and 2021 (spring vigor only). Spring vigor was recorded using a scale from 0–20, with 20 indicating no visible damage and 0 indicating mortality. Heading date was recorded as the date in which panicles were observed on at least 50% of an individual's tillers.

### Progeny performance experiment

A small population of progeny derived from the primary experiment were evaluated as part of a trial to measure yield performance in row plots conditions. Individuals within the rows were used to estimate genomic prediction accuracy. Open-pollinated seed from eight pseudo-F_2_ individuals which survived three winters from the primary experiment (described above) were planted as a progeny population in a row-plot trial constructed from greenhouse grown seedlings. The progeny seed was planted alongside half-sib families selected from the Liberty cultivar (9 families), a lowland population (1 family), and upland families (8 families). Each comparison family was the result of multiple selection cycles for late flowering or strong winter survival.

Within each family, seedlings were randomly assigned to family rows (30 cm between plants, 90 cm between rows; 15 individuals per row) and rows were assigned using an incomplete block design. Uneven germination and seed quantities resulted in an unbalanced design among the genotypes. Due to strong germination, the bulked progeny seedlings were used as a check family and was assigned to each incomplete block. Therefore, the progeny population was replicated 26 times, while the other families were replicated a mean of 5.6 times. The validation nursery was planted at Arlington, WI in May 2020. All plants and plots were allowed to grow during the establishment year and biomass was removed after killing frost. No fertilizer was applied. Plots were harvested with a flail chopper and plot weights determined by a load cell. Biomass harvest occurred during November 2021 and dry matter adjustment was based on three dry matter samples collected on the same date (∼500 g fresh weight each).

Heading date and winter survival was also measured on individual plants during the spring and summer of 2021. Winter survival scores were collected on 366 progeny individuals. Heading date was collected on 175 progeny individuals. Row-plot biomass yield best linear unbiased estimates (BLUEs) were calculated in a mixed-model with incomplete blocks as a random effect and genotypes as a fixed effect. Post hoc means comparison was carried out using a Dunnett's multiple comparison test which compared all families to the progeny population.

### Primary experiment DNA extraction, sequencing, and bioinformatics

Leaf samples were collected from all individuals after establishment in 2018. In 2019, a subset of the nursery was extracted for sequencing based on observed segregation for winter survival during spring 2019 and sufficient sample sizes within families. This resulted in genetic data from 18 pseudo-F_2_ families (n = 1,013), 17 F_1_ families (n = 618), 18 Founder individuals, and 23 F_1_ parents. The Founder individuals were deeply sequenced (targeting 40 reads per site), while the F_1_ and pseudo-F_2_ individuals were shallow sequenced (∼1–5 reads per site).

Sequencing data were generated at the DOE Joint Genome Institute using an Illumina NovaSeq S4 platform. Briefly, plate-based DNA library preparation for Illumina sequencing was performed on the PerkinElmer Sciclone NGS robotic liquid handling system using Kapa Biosystems library preparation kit (Roche). Next, 200 ng of sample DNA was sheared to 500 bp using a Covaris LE220 focused ultrasonicator. The sheared DNA fragments were size selected by double-SPRI and then the selected fragments were end-repaired, A-tailed, and ligated with Illumina compatible sequencing adaptors from IDT containing a unique molecular index barcode for each sample library.

The prepared libraries were then quantified using KAPA Illumina library quantification kit (Roche) and run on a LightCycler 480 real-time PCR instrument (Roche). The quantified libraries were then multiplexed and the pool of libraries was prepared for sequencing on the Illumina NovaSeq 6,000 sequencing platform using NovaSeq XP v1 reagent kits (Illumina), S4 flow cell, following a 2 × 150 indexed run recipe.

The program BBDuk (version 38.87) was used to remove contaminants, remove adapter sequences and trim reads where quality drops below 6 (Bushnell *et al.* 2014). Marker calling was carried out by aligning FASTQ reads using bwa-mem 0.7.17. Any PCR duplicates were marked using Picard tools. Alignment statistics were estimated using Samtools 1.9 and VCFs generated for each sample using Samtools mpileup (V 1.9) and VarScan.v2.4.3. Multi-sample VCFs were created after filtering for polymorphisms using bcftools-1.9.

To compensate for shallow sequencing within pseudo-F_2_ individuals, haplotype maps were assembled. This was carried out independently within each large family by creating a subset of bi-alleleic markers with both contrasting homozygotes within the ancestral Founder individuals and sufficient read depth in the F_1_ parents. Using this marker subset, a sliding window (100 sites) counting reads from either grandparent was used to assign ancestral probability within each pseudo-F_2_. Specifically, for each position parental calls were made with probability <0.1 or >0.9 assigned as homozygote of the given Founder ancestor and probabilities >0.2 and <0.75 assigned as heterozygotes. Run-length equivalents of parentage calls were calculated. Then assigned calls were decoded into haplotype breakpoints and short runs of heterozygosity between two homozygous regions were dropped. Specifically, short runs of <100 sites were dropped with recalculation of run-length equivalents to construct a final haplotype map. Individuals were removed from the haplotype map if they contained greater than 85% heterozygosity (n = 8) or only contained haplotypes from only one parent (n = 21). These individuals are most likely the result of pollen contamination.

### Progeny evaluation DNA extraction and sequencing

Leaf samples were collected for sequencing from 52 individuals within the progeny population. Since the identification of outliers is the most critical goal of genomic selection, a sampling method was used increase the incorporation of outlier individuals in the validation set. Specifically, individuals were sampled using weights from an inverted density distribution of the population's mean Z-scores. The mean Z-scores were calculated from each individual's heading date and winter survivorship scores. This resulted in a subset of the population with trait values slightly oversampled from the tails of the gaussian distribution. Genotyping by sequencing occurred on an Illumina sequencer (NovaSeq 6000) through the University of Wisconsin Biotechnology Center using PstI-MspI restriction enzyme digestion before ligating fragments to barcoded adaptors prior to polymerase chain reaction amplification. Data analysis of sequencer output used TASSEL (Glaubitz *et al.* 2014). Briefly, the barcoded sequence read outputs were collapsed into a set of unique sequence tags with counts. Tags were aligned to the reference genome (*P. virgatum* v5.1), assigning each tag to a position with the best unique alignment. The occupancies of tags for each sample were observed from barcode data. Resulting files were used to call single-nucleotide polymorphism markers (SNPs) at the tag locations on the genome, resulting 1,072,642 SNPs.

### Marker imputation and filtering

Because of the difference in sequencing platforms between the primary experiment and progeny population, the methods described below were run in parallel with and without the progeny samples included. The former SNP set was used for the variance analysis and genomic prediction cross-validation, and the latter was used for progeny prediction. For the primary data set, markers were filtered for the percentage missing sites (<20%), minor allele frequency (<0.05), and linkage disequilibrium (<0.90 with a marker within the nearest 15 variant sites) within the Founder individuals. The analysis with the progeny used a less stringent minor allele frequency (<0.025) to maximize the number of overlapping sites between the two sequencing runs. These initial filters resulted in 365,996 markers within the primary marker array and 204,682 with the array including progeny. To supplement shallower sequencing within the F_1_ individuals, missing markers were assigned where the resulting allele state is unambiguous (matching or contrasting homozygotes among the parents). Next, imputation of the remaining sites was carried out using the expectation-maximization algorithm (A.mat function, “rrBLUP” R package; Endelman 2011). This imputed data set was then assigned to pseudo-F_2_ individuals based on the haplotype map. Sites with fewer than 20% calls within the haplotype map were removed and a second round of imputation was used on the sites missing from the haplotype map. This second round of analysis and filtering resulted in 311,776 markers within the primary experimental population and 99,367 within the progeny data set.

### Quantitative genetic analysis

Variance estimation was carried out for heading date and winter survival scores using two single-trait models and a multi-trait model. The following model was used:


$$y\;=\;Xb\;+\;Zu_{a}\;+\;Zu_{d}\;+\;Zu_{e}\;+\;Zu_{R}+e$$

where y is the vector of phenotypes, b is the vector of fixed effects (year, fall vigor, and the interaction), **Z** represent the incidence matrix for individual genotypes, *u_a_, u_d_, u_e_,* and *u_R_* vectors represent the additive, dominance, epistatic and residual genetic effects respectively. The *e* represents a vector of the residuals. Variance structures are *u_a_* ∼N(0, **G** σ_a_^2^), *u_d_* ∼N(0, **D** σ_d_^2^), *u_E_* ∼N(0, **E** σ_E_^2^), *u_R_* ∼N(0, **I** σ_R_^2^) and *e* ∼N(0, **I** σ_e_^2^), where σ_a_^2^, σ_d_^2^, σ_E_^2^, and σ_R_^2^ are the additive, dominance, epistatic, and residual genotypic variance. The matrices **G**, **D**, and **E** are the realized additive relationship matrix, realized dominance relationship matrix, and realized epistatic relationship matrix. The matrix **I** is an identity matrix and used for isolating residual genotypic effects. Relationship matrices were derived from marker data using the R package *sommer* (Covarrubias-Pazaran 2016). The matrix **I** allows independent error variances between measurement years. For multi-trait analysis, heading date and winter survival score BLUPs were predicted simultaneously assuming unstructured covariance between traits.

Reliability was estimated for each trait and model. Reliability was calculated as:


$$r_{\;i}^{\;2}\;\;=\;1-\frac{\;Vg_{i}^{\;BLUP}}{{Vg}_{i}}$$

where $\;Vg_{i}^{\;BLUP}$ is the prediction error variance of an individual *i* (from the diagonal of the **C**_22g_ matrix), and ${Vg}_{i}$ is the **G** matrix diagonal (Schmidt *et al.* 2019). This statistic is comparable with heritability but is calculated on a genotype-difference basis (Schmidt *et al.* 2019).

For simplicity, genomic predictions were based on the above model with only major genetic effects (additive and residual) included. Genomic prediction was evaluated by cross-validation among families and by prediction of a generation of progeny individuals. Cross-validation used a two-stage process, where BLUEs were generated from a fixed effect model and BLUEs were then used in a model which accounted for relatedness between genotypes to predict breeding values for heading date and winter survivorship. The second model included weights based on the inverse of the square root of their standard error from the BLUE model (Cullis *et al.* 1996; Schulz-Streeck *et al.* 2013). The BLUPs were generated from the additive predicted breeding value of individuals. The residual genotypic variance was included in the prediction models because the variable predicted a meaningful proportion of variance and resulted in a superior model based on Akaike information criterion score.

Three cross-validation methods were used to assess model performance. This included masking half of a single family, masking and entire family, and masking the entire pseudo-F_2_ generation. Cross-validation predictive ability was calculated as the correlation between BLUPs from a complete model and BLUPs with a subset of field observations masked. Cross-validation masked either 50% or 100% of all pseudo-F_2_ or F_1_ families with greater than 40 individuals (n = 9).

Progeny predictive ability was evaluated using individuals from the progeny performance experiment (n = 52) for both winter survivorship scores and heading date. For progeny prediction, an additional set of predictions was carried out integrating epistatic variance and dominance variance for winter survivorship score prediction and heading date prediction, respectively. In addition, predictive ability of the progeny set was evaluated by masking all pseudo-F_2_ individuals to estimate the erosion of predictive ability when training data is not updated.

## Results

### Summary of field data

**Fig. 1.**
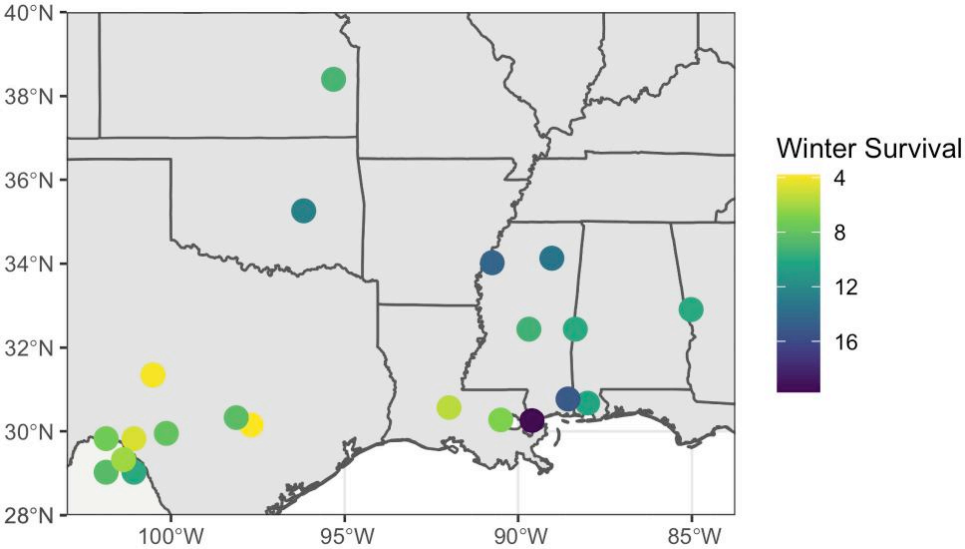
The approximate collection locations of founder populations used in crosses. Two populations with no geographic information were not included on the map. Color indicates the mean winter survivorship scores for the individuals collected at the location.

Overall, winter survivorship in Arlington, WI, was variable between and within families. Within the Founder individuals, mean winter survivorship ranged from 0 to 20 (Fig. 1). The Tolerant 1 and Tolerant 2 individuals had mean winter survivorship scores of 8.5 and 17.7, respectively. Populations from Texas had overall mean winter survivorship scores of 1.4 (maximum 8.1). Populations from Mexico had overall mean winter survivorship scores of 1.0 (maximum: 8.5). One collection site from Mississippi contained three individuals and all had mean winter survivorship scores of 20 (Fig. 1). Each of these individuals also flowered 15 days earlier than the next earliest Founder individual and likely represent a coastal ecotype population. Outside of this single outlier location, the mean winter survivorship scores for the Mississippi populations was 7.3. The three individuals from the Kanlow cultivar had mean winter survivorship scores of 11.3. Three individuals from the Kansas population had mean winter survivorship scores of 6.7.

Winter survivorship scores were collected for 3,158 individuals, 1,306 of which were re-sequenced. Since heading date measurements required at least 1 year of survival, only 1,458 individuals were measured for heading date, 609 of which were re-sequenced. During the initial winter (2018–2019), the F_1_ families sustained 48% mortality and the pseudo-F_2_ families sustained 66% mortality. During the second winter (2018–2019), mortality among the remaining individuals dropped to 9% for F_1_ families and 16% for the pseudo-F_2_ families. During the final year of measurements, mortality rate was 10% for F_1_ families and 12% for pseudo-F_2_ families.

**Fig. 2.**
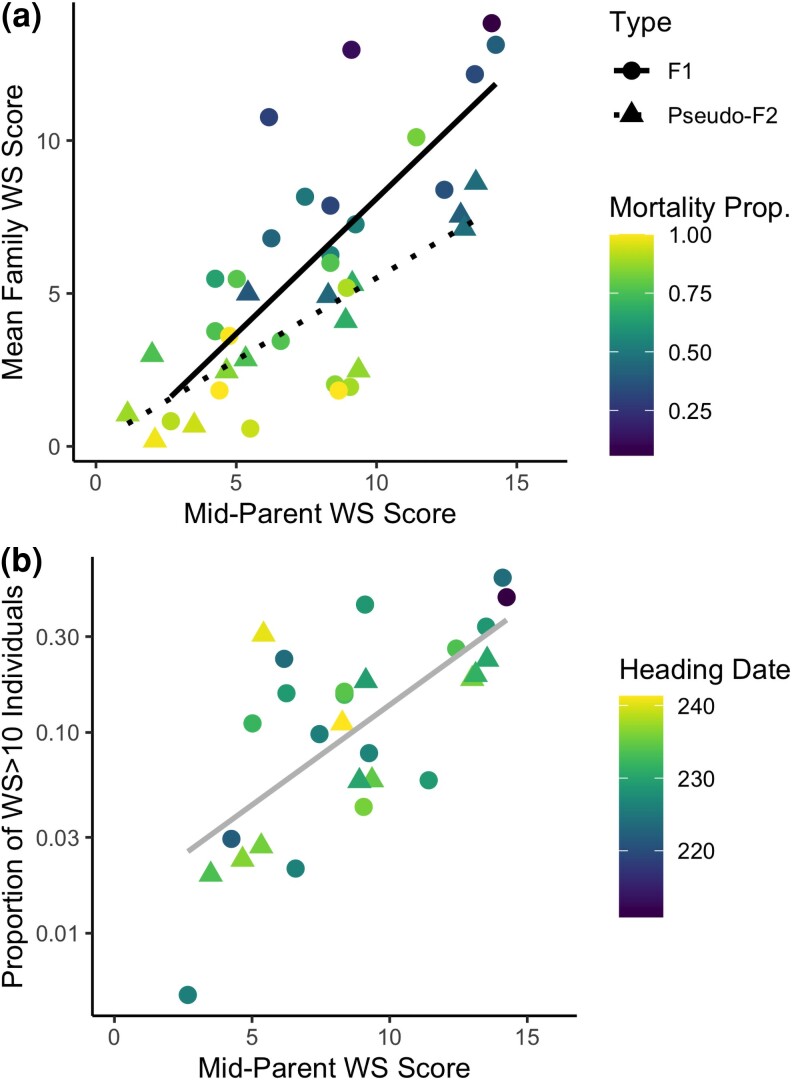
Scatterplots of switchgrass families depicting (a) the mean family winter survivorship (WS) scores and (b) the proportion of individuals with WS scores greater than 10 in the third year (note logarithmic y-axis) vs the mid-parent winter survivorship (WS) scores. The color of each data point indicates (A) the proportion of individuals which were deceased after 3 years and (B) the mean heading date of individuals in each family. Only families which contained greater than 10 individuals were included. Lines indicate linear regressions against the mid-parent WS score. Parent–progeny heritability within plot A was 0.71 overall, 0.70 within F_1_ families, and 0.88 within pseudo-F_2_ families.

The mean winter survivorship scores, indicating spring vigor or degree of survival, in 2019 were 6.9 for F_1_ families and 2.9 for pseudo-F_2_ families. In the spring of 2020, mean survivorship scores were 3.7 for F_1_ families and 1.3 for pseudo-F_2_ families. In the spring of 2021, mean survivorship scores were 7.1 for F_1_ families and 7.2 for pseudo-F_2_ families. Overall, a strong parent–progeny relationship was observed, with a winter survivorship mid-parent regression narrow-sense heritability of 0.71 (Fig. 2). Within the F_1_ families mid-parent regression heritability was 0.70, and mid-parent regression heritability was 0.88 within pseudo-F_2_ families.

The mean heading date was 227 ordinal DOY with a standard deviation of 10 days (Table 1). An overall phenotypic correlation of *r* = −0.32 was observed between winter survivorship and heading date (Supplementary Table 1). Similar to winter survivorship, narrow-sense heritability based on mid-parent regression was 0.64 overall, 0.54 within F_1_ families, and 0.74 within pseudo-F_2_ families.

**Table 1.** The variance estimates for models of winter survivorship scores and heading date (n = 1,306 and n = 609 with 3 and 2 years of data, respectively).

	Winter survival score		Heading date	
	Variance	SE	Variance	SE
Additive σ_g_^2^	12.52	2.6	25.38	7.3
Dominance σ_g_^2^	0	1.01	1.87	3.3
Epistatic σ_g_^2^	1.23	0.73	0	1.3
Residual σ_g_^2^	7.44	1.29	14.12	3.7
Residual 2019	25.52	1.4	50.91	3.9
Residual 2020	22.16	1.33	39.98	3.3
Residual 2021	20.5	1.64	-	-

**Fig. 3.**
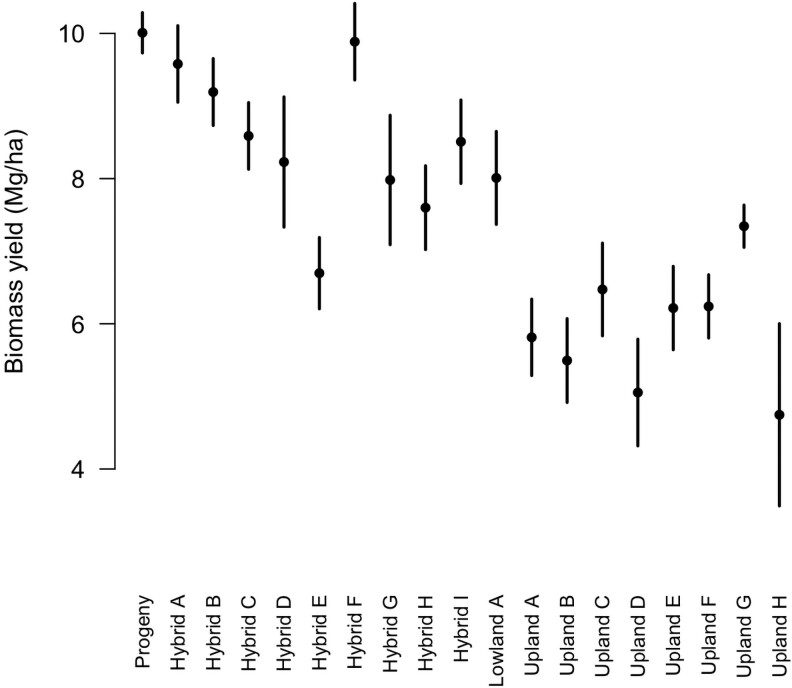
The yield performance of a row-plot trial containing half-sib families undergoing breeding and pooled progeny seed from eight pseudo-F_2_ individuals from the current experiment (progeny). Hybrid families (A–I) are half-sib families selected from late flowering winter tolerant individuals from the Liberty cultivar. The Lowland A family is from an early flowering Coastal ecotype individual. Upland families are either the result of three cycles for late flowering (A–F) or are unimproved Upland germplasm from the mid-Southern United States (G–H). Error bars represent the 95% confidence intervals around the best unbiased linear estimates.

The mean progeny winter survival score in 2021 was 12.5, with only one deceased individual observed in spring 2020 (0.2% of observations). The mean heading date was 210 DOY (range = 197 to 222 DOY). The phenotypic correlation between heading date and winter survivorship scores was *r* = −0.15 within the progeny. Dry biomass yield BLUEs within the progeny evaluation row plots ranged from 4.75 Mg ha^−1^ to 10.19 Mg ha^−1^, with the largest value representing the bulked progeny seed population (Fig. 3). The correlation between population heading date BLUEs and yield BLUEs in the progeny study was *r* = 0.85, with a linear regression slope of b = 0.19 Mg ha^−1^ d^−1^ (*P* = 0.002; Supplementary Fig. 1). A post hoc Dunnett's test indicated that all upland families and one hybrid family (Hybrid E, Fig. 3) had significantly lower yield relative to the progeny population.

### Winter survival and heading date variance is additive and moderately to highly reliable

In the single-trait model, genetic variance for winter survivorship was primarily additive, with moderate residual genetic variance, no dominance variance, and a small degree of epistatic variance (Table 1). Mean reliability was 0.76, with means of 0.66, 0.76, and 0.80 within the Founder, F_1_ and pseudo-F_2_ populations, respectively. In the single-trait model, genetic variance for heading date was primarily additive, with moderate residual genetic variance, no epistatic variance, and a small amount of dominance variance. Mean reliability was 0.68, with means of 0.50, 0.70, and 0.62 within the Founder, F_1_ and pseudo-F_2_ populations, respectively. In the multi-trait model, variances were similar to those from the single-trait models (Table 2). Mean reliability for winter survivorship was markedly lower than the single-trait model (0.65). Mean reliability for heading date was similar to the single-trait model (0.69).

**Table 2.** The variance estimates of a multi-trait model of winter survivorship score and heading date assuming unstructured covariance between traits (n = 609 and 2 years of data). The model including dominance variation was singular.

	Winter survival score		Heading date	
	variance	SE	Variance	SE
Additive σ_g_^2^	7.55	2.68	24.64	7.2
Epistatic σ_g_^2^	1.53	1.36	0.12	0.4
Residual σ_g_^2^	4.5	1.72	14.28	3.7
Residual 2019	51.4	3.92	32.28	2.3
Residual 2020	39.05	3.25	25.01	1.9

### Genomic prediction has high predictive ability within lowlands

Broadly, genomic predictive ability of winter survivorship was high (Table 3). Mean predictive ability through cross-validation within large families was 0.73 when sibling observations were included in the training data (50% masked, Table 3). Mean predictive ability was greater for pseudo-F_2_ relative to F_1_ families (0.88 and 0.63, respectively). When whole families were removed from the dataset, mean predictive ability was 0.52 and mean predictive ability was greater for pseudo-F_2_ relative to F_1_ families (0.66 and 0.41, respectively). When observations of the entire pseudo-F_2_ generation were removed from the training data and predicted, the mean predictive ability was 0.79. However, high predictive ability in this iteration was largely due to the accuracy associated with predicting the mean performance of families. The mean predictive ability within families when the entire pseudo-F_2_ generation was masked was 0.40.

**Table 3.** The cross-validated predictive ability of winter survivorship scores based on masking either 50% or 100% of a family. Predictive ability was the correlation coefficient of the genotypically estimated breeding values (GEBVs) and the best unbiased linear predictors (BLUPs) within each family. Bias is the slope between the GEBVs and BLUPs. Families with multiple rows indicate independent sibling mating events.

Family*^a^*	Type	n	50% Masked		100% Masked	
			Predictive ability	Bias	Predictive ability	Bias
Tolerant2×TX2-3	Pseudo-F_2_	136	0.73	0.67	0.27	0.22
MX5-2×Tolerant2	Pseudo-F_2_	155	0.87	0.81	0.62	0.61
MX5-2×Tolerant2	Pseudo-F_2_	49	0.98	1.15	0.97	1.10
MX5-2×Tolerant2	Pseudo-F_2_	37	0.90	0.84	0.90	0.92
Kanlow-2×MX2-1	Pseudo-F_2_	184	0.91	1.02	0.57	0.56
All Pseudo-F2		561	-	-	0.79*^b^*	1.01*^b^*
KS-1×Tolerant1	F_1_	213	0.73	0.40	0.44	0.12
MS1-2×Tolerant2	F_1_	115	0.70	0.46	0.66	0.28
MS1-3×MS3-3	F_1_	45	0.51	0.19	0.41	0.16
MS3-2×Tolerant1	F_1_	197	0.86	0.78	0.65	0.18
MX2-2×Tolerant2	F_1_	67	0.73	0.55	0.52	0.23
MX5-2×Tolerant2	F_1_	25	0.36	0.13	−0.02	−0.01
Tolerant2×TX2-1	F_1_	60	0.52	0.21	0.27	0.12

TX = Texas, MX = Mexico, MS = Mississippi, KS = Kansas.

Mean within-family predictive ability was 0.40. Mean within-family bias was 0.30.

Due to the smaller training dataset, the predictive ability of heading date was generally lower than winter survivorship (Table 4). Mean predictive ability within large families was 0.65 when sibling observations were included (Table 4). Mean predictive ability was slightly greater for pseudo-F_2_ relative to F_1_ families (0.70 and 0.63, respectively). When whole families were removed from the dataset, mean predictive ability was 0.53. Mean predictive ability was greater for pseudo-F_2_ relative to F_1_ families (0.58 and 0.51, respectively). Predictions of the entire pseudo-F_2_ generation resulted in a mean predictive ability of 0.74. Similar to observations for winter survivorship, the mean predictive ability within families was substantially lower when the entire pseudo-F_2_ generation was predicted (0.26).

**Table 4.** The cross-validated predictive ability of heading date based on masking either 50% or 100% of a family. Predictive ability was the correlation coefficient of the genotypically estimated breeding values (GEBVs) and the best unbiased linear predictors (BLUPs) within each family. Bias is the slope between the GEBVs and BLUPs.

Heading date	Type	n	50% Masked		100% Masked	
Family ^*^a^*^			Predictive ability	Bias	Predictive ability	Bias
Tolerant2×TX2-1	Pseudo-F_2_	44	0.81	1.00	0.67	0.64
MX5-2×Tolerant2	Pseudo-F_2_	35	0.58	0.39	0.48	0.28
All Pseudo-F_2_		92	-	-	0.74*^b^*	0.46*^b^*
KS-1×Tolerant1	F_1_	178	0.77	0.36	0.48	0.09
MS1-2×Tolerant2	F_1_	107	0.38	0.10	0.28	0.04
MS1-3×MS3-3	F_1_	26	0.72	0.34	0.70	0.39
MS3-2×Tolerant1	F_1_	121	0.83	0.57	0.63	0.22
Tolerant2×TX2-1	F_1_	41	0.47	0.33	0.46	0.29

*T*X = Texas, MX = Mexico, MS = Mississippi, KS = Kansas.

*M*ean within-family predictive ability was 0.26. Mean within-family bias was 0.09.

Progeny winter survivorship scores had a predictive ability of 0.71. With the pseudo-F_2_ families removed from the training data, the predictive ability unexpectedly increased to 0.73. A model including epistatic genetic variance resulted in a predictive ability of 0.72. Predictive ability for heading date within the progeny population was 0.53. A model including dominance genetic variance resulted in a predictive ability of 0.56. If the pseudo-F_2_ families were removed from the training data, the predictive ability increased slightly to 0.55. A post hoc analysis found that the inverted density sampling regime used for progeny population selection could inflate predictive ability by approximately 8% (reanalysis of Tilhou and Casler 2022a; unpublished data). This was not due to improved model performance, but appeared to be the result of predictive ability calculations with an excess number of individuals with trait values in the tails in the initial trait distribution.

Strong predictive ability could be an artifact of pedigree constructed in this study. Specifically, with only two major genetic donors of winter survivorship in the study (Tolerant1 and Tolerant2), winter survivorship could be proportional to the percentage of ancestry from a tolerant founder. A post hoc examination found significant correlations between winter survivorship scores of an individual and the genetic distance of that individual to the tolerant founders. The mean genetic distance between the progeny population (n = 52) and two tolerant founders was negatively correlated with progeny winter survival scores (−0.64). However, the relationship between genetic distance from tolerant founders and winter survivorship BLUPs was comparable witho the pseudo-F_2_ families which were not derived from a cross involving a tolerant parent (−0.63).

## Discussion

### Rapid improvements in winter survivorship can be made among diverse populations

Simply as a survey of biological adaptability, this study highlights how polyploid grasses can rapidly adapt to new environments through strong within-family segregation (Rieseberg *et al.* 1999). Despite moving three or four hardiness zones north of their initial environment (Fig. 1), many full-sib families produced progeny capable of surviving for three winters (Fig. 2). Intuitively, crosses which used one of the two winter tolerant founders resulted in greater improvements in survival (not presented). These results reinforce the conclusion that many southern populations contain traits which can confer winter survivorship and this aligns with previous reports of breeding progress within multiple parallel collections (Poudel *et al.* 2020).

Agronomically, this study shows that winter survivorship is highly heritable and promising lowland populations can be rapidly adapted to the north-central United States. Using the breeder's equation (Lush 1943; Δ*G*=σ_a_*ir*), one can calculate the expected gain from selection using the narrow-sense heritability for winter survivorship (r^2^ = 0.71, from parent-offspring regression in Fig. 2), the estimated additive variance (σ_a_^2^ = 12.5; Table 1), and selection of the top 10% of a population (i = 1.75). Theoretically, these variables predict an improvement of 5.2 points in winter survivorship scores per selection cycle. Of course, the exact rate of gain is difficult to extrapolate since winter survivorship scores do not represent a linear biological trait.

In practice, survivorship improvement could be further accelerated during early winter mortality events by increasing population size and selection intensity. Growing hundreds or thousands of plants to acquire tolerant individuals is feasible, particularly if individuals can be grown in dense seeded sod, which is a more accurate representation of commercial production conditions (Tilhou *et al.* 2022). This strategy, combined with gradual movement of material into harsher, northern sites, could maintain strong selection pressure during field evaluations (Poudel *et al.* 2020). The ability to rapidly adapt southern lowland populations to northern regions opens up many new breeding opportunities, since the lowland ecotype includes the center of switchgrass diversity along the United States Gulf Coast (Zhang *et al.* 2011; Evans *et al.* 2018).

### Partial inbreeding may be a tool to accelerate switchgrass breeding progress

Sibling mating was adopted in this study to attempt to isolate genetic regions that could confer winter survivorship, but these results indicate that sibling crosses could be a useful breeding tool. In the current project, this method increased winter mortality in moderately tolerant populations. More broadly, however, it shows the feasibility of producing weakly-inbred lines of switchgrass. Switchgrass self-pollination is rare and unpredictable (Liu and Wu 2012). Therefore, sibling mating could provide an alternative method which could facilitate study of switchgrass heterosis.

In the current study, the pseudo-F_2_ families were visually shorter than their F_1_ relatives and prone to greater winter mortality (Fig. 3). Similar inbreeding depression has been reported switchgrass and is a genetic outcome consistent with its outcrossing reproductive habit (Chang *et al.* 2022; Casler and Lee, personal communication). Since inbreeding depression tends to co-occur with heterosis (Mackay *et al.* 2021), these results suggest that further progress can be made through yield heterosis in switchgrass (Vogel and Mitchell 2008; Shrestha *et al.* 2021; Edmé and Mitchell 2021).

### Is late flowering and winter tolerant switchgrass possible?

There have been observations of a strong antagonistic relationship between winter survivorship and late flowering (Schwartz and Amasino 2013). This relationship is a challenge because flowering date is positively correlated with biomass yield (Supplementary Table 1, Supplementary Fig. 1; Casler 2019; Tilhou and Casler 2022b). Interestingly, the strength of this relationship decreased across the generations evaluated in this experiment, from *r* = −0.60 among the Founder individuals to *r* = −0.15 among progeny individuals. A portion of this erosion may be due to the elimination of late flowering individuals. However, it is likely that the degree of genetic linkage is also being reduced. Due to the magnitude of linkage reduction across only three generations, a large proportion of previously observed genetic linkage was due to population structure. Since population structure can be rapidly reduced, this is promising evidence that tradeoffs between winter survivorship and yield will be minor. There is prior evidence that flowering is linked to nutrient remobilization and hardening in switchgrass (Schwartz and Amasino 2013; Sarath *et al.* 2014). This assumption is, most likely, broadly true for the species in the wild. However, selected switchgrass individuals could prepare for winter in response to photoperiod, rather than flowering per se. If this is possible, then full senescence is not biologically necessary for winter survival and prioritizing further extensions of vegetative growth would be valuable for biomass production. For example, most of this germplasm originated in the southern United States, and clearly produces greater biomass production than locally derived populations (Poudel *et al.* 2020). The southern limit of the switchgrass native range is near the Mexico-Guatemala border. Therefore, it is conceivable that genotypes from still further south from the current collection region could provide long periods of vegetative growth and greater biomass accumulation.

Alternatively, it is possible (even likely) that unseen damage or nutrient loss is occurring due to excessively late flowering, and that late flowering genotypes will have poor vigor in commercial sward conditions. In the progeny row-plot evaluation, two families derived from the Liberty cultivar produced comparable biomass yield to the lowlands despite earlier flowering time (Fig. 3; Supplementary Fig. 1). Future research is needed on this topic. From a breeding perspective, additional selection effort for vigor in late flowering genotypes may be necessary even after acceptable survivorship rates are obtained. It is likely that the ability to produce reliable long-term biomass requires genetic improvements beyond what is required for mere survival.

### Genomic prediction for winter survivorship was accurate but appears unnecessary

Predictive ability of winter survivorship using genomic selection was relatively strong and this level of predictive ability would result in reliable genetic progress with field evaluations occurring only as needed to recalibrate after one or two generations of selection (Tables 1 and 2). With this level of precision, recurrent genomic selection may only require a small number of selection cycles to generate a robust population, but the exact rate of gain is difficult to extrapolate since winter survivorship scores are arbitrary visual measurements. Although genomic selection is accurate, a simple pedigreed breeding program could result in comparable progress if sequencing is unavailable or cost prohibitive. Therefore, genomic selection will be most valuable as an additional target of selection if sequencing is already being carried out for a complex trait such as biomass yield (Simeao Resende *et al.* 2014).

Progeny predictive ability was not reduced when the entire pseudo-F_2_ family generation was omitted, which was a surprising result. Usually, genomic prediction performance is superior then the population being predicted is closely related to the training data. This results in a penalty in predictive ability when multiple cycles of selection and recombination are carried out without updating training data (Neyhart *et al.* 2017). Therefore, this result suggests that only minor reductions in predictive ability occur when predictions are made across multiple generations. It is possible that the pseudo-F_2_ families provided poor training data and their omission improved model performance but this is unlikely. Reliability was comparable between the F_1_ and pseudo-F_2_ families for winter survivorship (0.76 vs 0.80, respectively), and only a minor decrease was observed between F_1_ and pseudo-F_2_ families for heading date reliability (0.70 vs 0.62, respectively).

Alternatively, this strong and persistent predictive ability could be an artifact of the population structure generated in this study. Specifically, winter survivorship was proportional to the overall percentage of ancestry from a tolerant founder. This correlation was strong within progeny validation, but not as strong as the predictive ability obtained through genomic prediction (−0.62 and 0.71, respectively). This result is not surprising since this experiment utilized the GBLUP model, which relies heavily on genetic relationships for prediction (VanRaden 2008). Overall, this strong predictive ability indicates that individual sequencing may be an overly resource-intensive prediction strategy. Instead, a moderate number of rapid morphological markers may have sufficient predictive ability for winter survivorship selections. This strategy has been referred to as phenomic selection (Rincent *et al.* 2018). Further research would be needed to evaluate best-practices in phenomic trait prediction in switchgrass, but promising results have been reported using near-infrared spectroscopy, which is already used for biomass quality traits (Lane *et al.* 2020).

## Conclusion

This study described the winter survivorship and survival of multiple lowland switchgrass families across three years in the North-Central United States and found that genetic variance for winter survivorship is largely additive, has high narrow-sense heritability (0.71) and reliability (0.76). Heading date, a potential covariate for winter survivorship, had similarly high reliability (0.68), but a multi-trait model including heading date did not improve winter survival predictive ability. Further, the genetic correlation between heading date and winter survivorship appeared to erode across multiple recombination events. In a single-trait model, genomic predictive ability was generally high, even with large portions of the dataset omitted. Despite these promising results for genomic prediction, phenotypic selection successfully isolated winter tolerant genotypes from multiple crosses of different backgrounds and may continue to be the most efficient selection strategy to develop high-biomass and sustainable switchgrass cultivars. Instead, genomic prediction of winter survival will be applicable in populations already being sequenced for more complex traits, such as biomass yield.

## Supplementary Material

jkad014_Supplementary_Data

## Data Availability

Raw reads for this study are available in the Sequence Read Archive database (https://www.ncbi.nlm.nih.gov/sra; associated data included in supplementary files). Field data and progeny marker data in variant call format is available through Dryad Digital Repository (https://doi.org/10.5061/dryad.2jm63xss7). Field data and pedigree information are included as supplementary files. To aid in replication, R code used for analysis is also attached as Supplementary supplementary data. Supplemental material available at G3 online.
