# Supplementary material for: Genomic prediction of switchgrass winter survivorship across diverse lowland populations
Source: G3 (Bethesda). 2023 Jan 17;13(3):jkad014. doi: 10.1093/g3journal/jkad014 (PMC9997553; doi:10.1093/g3journal/jkad014)
Supplement: jkad014_Supplementary_Data [file jkad014_supplementary_data.zip › Figure_S1_and_Table_S1_G3-2022-403803.docx]

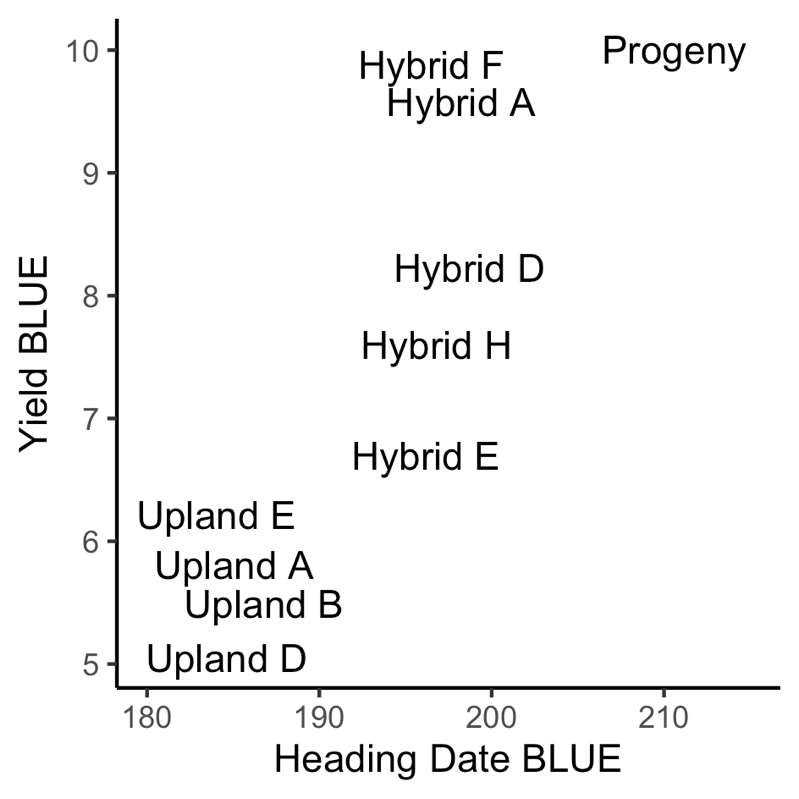


Figure S1: A scatterplot of populations evaluated for row-plot yield (Mg ha^-1^) and heading date.

Table S1: Phenotypic correlations among winter survivorship scores and heading dates across measurement years.

|  | Winter Survival Score (2019) | Winter Survival Score (2020) | Winter Survival Score (2021) | Heading Date (2019) | Heading Date (2020) |
| --- | --- | --- | --- | --- | --- |
| Winter Survival Score (2019) | 1 |  |  |  |  |
| Winter Survival Score (2020) | 0.74 | 1 |  |  |  |
| Winter Survival Score (2021) | 0.28 | 0.51 | 1 |  |  |
| Heading Date (2019) | -0.51 | -0.24 | -0.11 | 1 |  |
| Heading Date (2020) | -0.10 | -0.43 | -0.30 | 0.45 | 1 |
